# American Dental Convention—Fourth Annual Session

**Published:** 1858-09

**Authors:** 


### Proceedings of Societies.

### AMERICAN DENTAL CONVENTION—FOURTH ANNUAL SESSION.

### MINUTES.

FIRST DAY, MORNING SESSION.

Convention assembled at the Melodeon, in the city of Cincinnati, on Tuesday morning, August 3d.

Called to order by the President, Dr. Taylor, at about 10 o'clock. Members present.

NEW HAMPSHIRE.—Dr. A. Blake, Sardinia ; Dr. Frank Fuller, Portsmouth.

VERMONT.—Dr. M. Tefft, West Poultney.

CONNECTICUT.—Dr. W. Potter, Norwich.

NEW YORK.—Dr. L. W. Rogers, Utica ; Dr. W. B. Roberts, New York City ; Dr. G. H. Perine, New York City ; Dr. John Allen, New York City ; Dr. S. F. Tremaine, Rome ; Dr. H. Jameson, Jr., Lyons.

PENNSYLVANIA.—Dr. Robt. Vandervert, Pittsburg ; Dr. J. Westbay, Pittsburg ; Dr. W. M. Wright. Pittsburg ; Dr. S. S. White, Philadelphia ; Dr. A. W. Asay, Philadelphia ; Dr. W. R. Greenlee, Meadville ; Dr. F. J. Chandler, Rochester ; Dr. J. B. Williams, Allegheny City; Dr. J. Murray, Beaver; Dr. J. McCalla, Lancaster.

VIRGINIA.—Dr. James Johnston, Staunton.

MISSOURI.—Dr. Charles Merry, St. Louis ; Dr. A. M. Leslie, St. Louis ; Dr. Isaiah Forbes, St. Louis ; Dr. S. Dunham, St. Louis.

GEORGIA.—D. S. Chase, Augusta.

MISSISSIPPI.—Dr. A. Berry, Raymond.

TENNESSEE.—Dr. B. Wood, Nashville ; Dr. W. H. Morgan, Nashville ; Dr. Bryan Cole, Memphis ; Dr. C. F. Munster, Jackson.

KENTUCKY.—Dr. J. B. Lindsay, Maysville ; Dr. H. Munro, Lebanon ; Dr. W. D. Stone, Lexington ; Dr. S. Driggs, Lexington ; Dr. D. Dougherty, Bardstown ; Dr. James Nourse, Cloverport ; Dr. S. Tuttle, Shelbyville ; Dr. J. W. Leatherman, Jeffersontown ; Dr. Rogers, Shelbyville ; Dr. H. McCollum, Augusta ; Dr. Geo. P. Newlin, Danville ; Dr. R. Peckover, Paris ; Dr. B. J. Dudley, Louisville ; Dr. J. W. Grant, Lancaster.

ILLINOIS.—Dr. W. W. Allport, Chicago; Dr. I. B. Branch, Galena; Dr. W. P. Kingsley, Freeport.

MICHIGAN.—Dr. P. S. Grimes, Kalamazoo; Dr. John Mansfield, Niles.

INDIANA.—Dr. David Cloud, Green Castle; Dr. David Marshall, Carthage; Dr. M. H. Shadoan, Rockport; Dr. J. P. Ulrey, Rising Sun; Dr. W. R. Webster, Richmond; Dr. S. M. Goode, Madison; Dr. J. Hood, Greensburg; Dr. John Keely, Brookville; Dr. George Lupton, Shelbyville.

OHIO.— Dr. C. Palmer, Warren; Dr. A. E. Lyman, Newton Falls, Dr. R. McDowell, Wooster; Dr. S. Brown, Piqua; Dr. Wm. B. Ingersoll, Oberlin; Dr. C. M. Kelsey, Mt. Vernon; Dr. George Watt, Xenia; Dr. S. Clippenger, Bellefontaine; Dr. W. H. Atkinson, Cleveland; Dr. J. Sanford, Chillicothe; Dr. H. A. Smith, Oxford; Dr. J. F. Siddall, Oberlin; Dr. J. Bercher, Findley; Dr. J. L. Dunlap, Chillicothe; Dr. Alfred Terry, Norwalk; Dr. F. R. Rehwinkle, Chillicothe; Dr. Wm. Hall, Piqua; Dr. C. Sill, Franklin; Dr. D. F. Dyche, Lebanon; Dr. Wm. E. Dunn, Delaware; Dr. E. Conway, Dayton; Dr. G. W. Keely, Oxford; Dr. D. W. Harris, West Liberty; Drs. Jas. Taylor, Chas. Bonsall, S. L. Hamlen, B. D. Wheeler, J. Richardson, J. Taft, G. L. Van Emmen, E. W. Ruth, Jas. Erwin, Cincinnati.

The records of the last annual session of the convention, were read by the Secretary and approved.

Dr. Taft, chairman of the committee appointed last year, to prepare business for this Convention, made the following report:

I. President's Address.
II. Election of Officers.
III. Report of Committees.
IV. Reading original papers and Essays.
V. Discussions:
    1. The best means of securing good teeth.
    2. The best means of preserving the teeth.
    3. Treatment of exposed nerves.
    4. Mechanical Dentistry.
    5. Exhibitions of Implements, etc.
    6. Filling Teeth.
    7. Miscellaneous.

Report adopted

The President presented a letter from the Vice President,

Dr. Tucker of Boston, giving his reasons for being absent, and expressing his regret, that he could not be present at this Convention, which was read and ordered to be placed on file.

The President then made a short and impressive address, which was well received.

The Convention then proceeded to the election of officers. On the first ballot for President there was no choice. Second ballot, Dr. Forbes of St. Louis received 29 votes, Dr. Allport of Chicago, 19. On motion of Dr. Allport, Dr. Forbes' election was then made unanimous.

Dr. Rogers then offered the following:

*Resolved*, That a Treasurer be appointed separate from the Recording Secretary, who shall, in addition to the usual duties, keep a record of the names of all the members of the Convention, and collect such assessment, as may be made upon each.

Adopted.

On the first and second ballot for Vice President, there was no choice. On the third ballot, Dr. Rogers received 28 votes, Dr. Allen, 25. On motion, the election of Dr. Rogers was made unanimous.

On motion,

*Resolved*, That the remaining officers be chosen, viva voce.

Dr. J. Taft of Cincinnati, was chosen Recording Secretary.
Dr. D. S. Chase of Augusta, Ga., Corresponding Secretary.
Dr. W. B. Roberts of New York, Treasurer.

On motion adjourned to 3 o'clock, P. M.

First Day, Afternoon Session.

Convention met pursuant to adjournment. Record of morning session was read and approved.

On motion, Drs. Allport and D. S. Chase, were appointed a committee, to conduct the President elect to the chair.

On taking the chair, the President made some appropriate remarks, (for which see report of discussions.)

On motion of Dr. Wright, of Pittsburg,

*Resolved*, That the thanks of this Convention be tendered to Dr. James Taylor, our retiring President, for the able and impartial manner in which he has presided over its deliberations, as well as for his spontaneous address on retiring, which has embraced so much of interest and value to the profession; and especially, for his remarks on the history and progress of dental education, in this country, during the past thirty years.

On motion of Dr. Perine, of New York,

*Resolved*, That the thanks of this Convention be tendered to the retiring officers, for the very faithful and efficient manner in which they have discharged their duties, during the past year.

On motion,

*Resolved*, That an executive committee of five, be appointed by the chair, to serve during the next year.

Committee—Drs. Allport of Chicago, Wright of Pittsburg, Leslie of St. Louis, Fuller of Portsmouth, N. H., and Stone of Lexington, Ky.

On motion of Dr. I. B. Branch,

*Resolved*, That the Treasurer be requested, if practicable, to procure a stenographic reporter, to serve this Convention, during its present session, and pay the same from the funds in the treasury.

On motion of Dr. J. Taylor,

*Resolved*, That in the opening of each subject for discussion, written remarks shall first be in order, and that the gentleman opening the discussion on any given subject, be permitted to close the same.

The executive committee were requested to examine the books and vouchers of the Secretary and Treasurer. After the examination they made the following report, viz:

We, your executive committee, having examined the vouchers of the Secretary for the past year, find the same correct, and report a balance remaining in the treasury, of $262.96.

W. W. Allport, W. D. Stone,
W. M. Wright, F. Fuller,
A. M. Leslie.

On motion of Dr. Wright,

*Resolved*, That when we adjourn, we adjourn to meet to-morrow morning at 8 o'clock, at the lecture room of the Ohio Dental College.

The first topic for discussion, viz: "The best means of securing good teeth," was now announced by the President, and papers pertaining to it called for. Drs. Richardson and Ingersoll, read interesting papers upon the subject; after which the subject was fully and ably discussed by Drs. Atkinson, Berry, Perine, Rogers, Wright and Watt.

Adjourned to meet to-morrow morning at 8 o'clock.

WEDNESDAY MORNING, AUGUST 4th. 8 O'CLOCK.

The Convention met according to adjournment, at the lecture room of the Dental College. The minutes of the preceding session, were read and adopted.

The discussion of the same subject was resumed; remarks made by Drs. Fuller, Roberts, Rehwinkle, Kelsey, Stone, Goode, Chandler, Taylor, Rogers of Cincinnati, Murray, DeCamp, McKellops, Dunham and Taft.

The second topic, "The best means of preserving the teeth," was taken up, and discussed by Drs. Watt, Taft and Taylor.

Adjourned to meet at same place, at 3 o'clock, P. M.

AFTERNOON SESSION.

The Convention met as by adjournment. The minutes of the morning session were read and approved.

The third topic, "Treatment of exposed nerves," was taken up, and discussed by Drs. Atkinson, Branch, Asay, Wright, Grant, Taft, Ingersoll, Johnston, Stone, Berry and others.

A dispatch was received by the President, announcing the death of Dr. C. O. Cone, of Baltimare, on the 1st of August.

Upon the announcement of the demise of Dr. Cone, on motion,

*Resolved*, That the Convention, as a mark of respect, do now adjourn.

Adjourned to meet same place, to-morrow, at 8 o'clock.

Dental College, Thursday Morning, 8 o'clock.

Convention met and was called to order by the President. The minutes of the preceding session, were read and approved.

Dr. Wright offered the following:

Whereas, It is the custom of the several railroad companies of the United States, to permit persons, passing to and from Conventions,—religious and secular—upon the payment of half fare; Therefore,

*Be it Resolved*, That the Executive Committee of this body, be authorized to represent our interests to the Presidents of the various railroad companies, in the matter of the aforesaid reduction of fare, so that members of the dental profession, who may desire to attend the Convention next year, may have the benefit of such arrangement, if made.

The topic last under discussion, was resumed by Drs. Forbes and Kelsey, when the discussion upon that subject was closed.

On motion of Dr. Perine,

*Resolved*, That we now decide upon the place for the next annual meeting of the Convention.

Dr. Perine suggested Buffalo, N. Y.; Dr. Taylor suggested Niagara Falls, N. Y.; Dr. Atkinson suggested Cleveland, O.

On motion, it was unanimously

*Resolved*, That when we adjourn, it be to meet on the first Tuesday of August, 1859, at Niagara Falls.

On motion of Dr. Allport,

*Resolved*, That a committee of one be appointed, to make all necessary arrangements for said meeting, and to report to the profession, previous to the meeting.

Committee—Dr. Rogers, Utica, N. Y.

Adjourned to meet at 2½ o'clock, P. M.

Afternoon Session.

Convention met at 2½ o'clock, P. M. Minutes of the morning session read and approved.

Topic No. 4, "Mechanical Dentistry," was called up for discussion.

Dr. Fuller described, by request, his method of making partial dentures, to be retained by atmospheric pressure.

Dr. J. Allen read a paper on "Artificial Dentures," (for which see Page 25, this No.) after which Dr. Roberts of New York, addressed the Convention upon the subject of continuous gum work.

On motion of Dr. Chase,

*Resolved,* That we now take up the subject of "Filling Teeth,"

Which was discussed by Drs. Atkinson, Allport, Stone, Rogers, Dunham, Branch, Tuttle, Wright, and others.

On motion of Dr. Taylor,

*Resolved,* That the discussion on filling teeth, be now closed, and that twenty minutes be appropriated to the discussion of "local anæsthesia,"

At the expiration of which time, "Miscellaneous Subjects" were taken up.

Dr. Wright described the treatment of a case of irregularity.

By Dr. McKellops,

*Resolved,* That the Convention appoint a committee of five, to memorialize Congress, on the necessity of appointing dentists for service in the regular army. The committee to act in concert with that of the "Western Dental Society"

Committee—Dr. James Taylor, Cincinnati, O.; Dr. Geo. Watt, Xenia, O.; Dr. Frank Fuller, Portsmouth, N. H.; Dr. W. H. Atkinson, Cleveland, O.; Dr. Stone, Lexington, Ky.

On motion,

*Resolved,* That the Treasurer be authorized to pay the Janitor of the Ohio Dental College, ten dollars, for his services to this Convention.

Dr. Allport presented a case, in which a full set of continuous gum teeth, worn by a lady, without any peculiarity of constitution, produced a peculiar and excessive disease of the entire buccal cavity. Dr. A. asked for information upon the subject, its cause, treatment, etc.

Dr. Rehwinkle described an interesting case of necrosis of the inferior maxilla.

Dr. Osmond described a case of soldering lower sets of teeth, particularly applicable to silver cases.

Dr. Rogers of Utica, N. Y., reported a case of diseased mouth, similar to that reported by Dr. Allport, by wearing a gold plate. Was unable to give any satisfactory definition of it.

Dr. Watt presented a surgical case from Dr. Allport, for which see report of discussions.

Dr. Vandervert described his method of welding platinum, and also gave a demonstration, welding some pieces of platinum, perfectly.

Adjourned to meet to-morrow morning at 8 o'clock.

FRIDAY MORNING, 8 O'CLOCK.

The Convention met at the College. The minutes of last meeting were read and adopted.

A paper was read by Dr. Murray of Pa., upon "Diseased Antrum;" ordered to be printed. Also, a paper was read by Dr. McCalla, on "Lateral Pressure of the Teeth, considered in relation to obscure Nervous Affection," which was requested for publication. (See present No. page 43.)

Cases of diseased antrum were described by Drs. Kelsey, Allen and Wright.

Case of irregularity by Dr. Wright.

A case of osseous tumor was described by Dr. Siddall of Oberlin, O.

A case of protrusion of the inferior maxilla was reported by Dr. McKellops, and suggestions called for in regard to the remedy.

On motion, the discussion upon filling teeth, was resumed for one hour ; remarks were made by Drs. Palmer, Rogers, Fuller and Forbes.

Dr. Atkinson introduced to the Convention, Dr. Brainard of Cleveland, who made some appropriate remarks.

Dr. Chandler described the treatment of an obstinate case of hemorrhage. Several members made remarks on hemorrhage.

The report of the Investigating Committee was read by

Dr. Taft, and adopted, and the committee requested to prosecute the enterprise.

Report of the Dental Investigating Committee.—Your Committee would most respectfully report,—that immediately after the appointment, the committee organized by selecting Dr. A. Blakesly of Utica, N. Y., Chairman; Dr. T. L. Buckingham of Philadelphia, Pa., Secretary; and Dr. J. Taft of Cincinnati, Treasurer.

Your Committee promptly issued some four thousand circulars, setting forth the aims and objects of the enterprise briefly, and soliciting the aid and co-operation of the profession, so far as they are disposed. These were sent to all the members of the profession in the United States, so far as the Committee could obtain their names and localities. Some of these were responded to encouragingly, promising co-operation, and making small remittances; all regretting that they could not now do more, and promising more in the future, if the enterprise was sustained. Many others wrote encouragingly, but acted discouragingly, or rather, we were discouraged because they did not act at all. All, however, had much to say in regard to the peculiar character of the times, financially considered, and we supposed that a much greater amount would have been appropriated, had there not been in fact, much reality in the cause assigned.

Your Committee also made some effort to find some person or persons, whose services could be commanded, to conduct experiments and investigations, as contemplated in the original plan. From the inquiry made, we think there would be no difficulty to procure such a person. The Committee were not fully warranted in making an engagement with any one, till the amount requisite for payment, could be commanded. We think this could have been done long before this, had not the financial troubles existed. The necessity for the prosecution of the work is now just as great as it has been at any time past, and your Committee is encouraged to believe, that the profession only require to know that the prosecution of the enterprise is a fixed fact, that they will sustain it. There has been pledged and paid over, $200. The expenses, some $60, have been paid out of this. Your Committee request direction from this body, as to the course to be pursued in future.

J. Taft, A. Blakesly, T. L. Buckingham.

An instrument called "Universal Spiral Spring Drill" was exhibited to the Convention, by Dr. Merry of St. Louis, who is the inventor. A vote of thanks was tendered to Dr. Merry. It was esteemed by all as a very valuable instrument. A cut and description is given on another page. It was requested that instruments of this kind be manufactured, and put into the market immediately.

A resolution of thanks was extended to the members of the profession, of Cincinnati, for their hospitality, manifested to the members attending the Convention.

A resolution of thanks was also extended to the Faculty of the Ohio Dental College, for the use of their room, for its sessions.

Adjourned to meet on the 1st Tuesday of August, 1859, at Niagara Falls.

J. TAFT, Secretary.

Dr. F. Fuller was appointed to report on Conway's "Dental Lamp and Furnace," and at an informal meeting, presented the following report:

*Resolved*, That the principle of Conway's patent "Lamp and Furnace," is correct, and that it may, in our opinion, be made highly valuable for dental purposes, by increasing the capacity of the furnace.

F. FULLER, Committee.

